# Microbial quality, instrumental texture, and color profile evaluation of edible by-products obtained from Barbari goats

**DOI:** 10.14202/vetworld.2015.97-102

**Published:** 2015-01-29

**Authors:** Pramila Umaraw, V. Pathak, V. Rajkumar, Arun K. Verma, V. P. Singh, Akhilesh K. Verma

**Affiliations:** 1 Department of Livestock Products Technology, College of Veterinary Sciences and Animal Husbandry, Uttar Pradesh Pandit Deen Dayal Upadhyaya Pashu Chikitsa Vigyan Vishwavidyalaya Evam Go Anusandhan Sansthan, Mathura, Uttar Pradesh, India; 2 Goat Products Technology Laboratory, Central Institute for Research on Goats, Makhdoom, Mathura, Uttar Pradesh, India

**Keywords:** edible byproducts, hunter color lab, microbial quality, textural characteristics

## Abstract

**Aim:**

The study was conducted to estimate the contribution of edible byproducts of Barbari kids to their live and carcass weight as well as to assess textural and color characteristics and microbiological status of these byproducts.

**Materials and Methods:**

Percent live weight, Percent carcass weight, Texture, color, and microbiological analysis was done for edible byproducts *viz*. liver, heart, kidney, spleen, brain and testicle and longissimus dorsi muscle was taken as a reference.

**Results:**

The edible byproducts of Barbari kids constitute about 3% of the live weight of an animal of which liver contributed maximum (1.47%) followed by testicles (0.69%) and heart (0.41%). While the same constituted 3.57, 1.70, and 0.99%, respectively on carcass weight. There was significant (p<0.05) difference among all organs regarding textural properties. Liver required the maximum shear force and work of shear (121.48N and 32.19 kg-sec) followed by spleen and heart. All organs revealed characteristics color values (L*, a*, b*, chroma, and hue) which were significantly different (p<0.05) from muscle values. The total viable count, coliform count showed slight differences for all organs studied. The staphylococcus counts were low with little differences among organs.

**Conclusion:**

Edible byproducts have a significant contribution to carcass weight which could enhance total edible portion of the carcass. Efficient utilization of these by-products returns good source of revenue to the meat industries. Textural and color analysis give information for their incorporation in comminuted meat products, and microbial study tells about the storage study. However, study was in the preliminary and basic step forward toward better utilization of 3% of live animal which could increase the saleable cost of animal by 6.94%.

## Introduction

Goats play a very vital role in the livelihood security of the small, marginal, and landless farmers especially in arid, semi-arid, and mountainous regions. Their popularity is mainly due to their short generation intervals, higher rate of prolificacy, adaptability, and relatively inexpensive rearing. They thrive well and reproduce in tropical, cold, humid as well as dry regions. India ranks first in goat population having 154 million goats [1]. The consumption of chevon has increased in past few years largely due to its unique flavor, lean characteristics and universal socio-religious acceptability [2]. But against the beef and pork, goat meat has regional preferences such as in African countries, Mediterranean countries, Latin America, Middle East and the Asian Southwest region. The majority of goat population is concentrated in the developing countries where due to religious or traditional customs goat meat or chevon consumption is quite high. Meat production is growing fast, especially in the countries of East and Southeast Asia [3]. Mahanjana and Cronje [4] in a cross culture-education-ethnic study highlighted that goat meat and African cultural activities have a close association. There is an increased demand of chevon in Greece, Italy, France, Spain, and Portugal around Christmas, Easter or any other festive season. The food consumption pattern of world is expected to increase in the livestock products sector driven by the growth rate of 4% in meat consumption by the developing countries [5]. While there would be very slight increase or a stagnant phase of growth in the developed countries regarding meat consumptions. At present, livestock sector has become the biggest competitor for utilization of agricultural land for human consumption utilizing about 30% of global arable land [6]. Environmental implications, as well as nutritional imbalance in diets, are pointing toward the sustainable livestock production with efficient utilization of each animal slaughtered for human consumption. Efficient utilization of the edible by-products can be one such way.

Edible byproducts constitute about 20-30% of live weight of the animal in case of cattle buffalo, sheep, and goats. It has been estimated that 11.4% of the gross income from beef and 7.5% of the income from pork can be generated from the utilization of by-products [7]. The hygienic handling and collection of these edible by-products plays a pivotal role in their efficient utilization. The microbiological status of these by-products during processing can be thus employed as an analytical tool for assessing hygienic collection and handling.

Meat processing industry is evidencing a boom because of the increasing interest of consumers in convenience foods. Convenience foods occupy about one-third of British food markets. US are the largest consumer of chilled ready-made meals followed by UK [8]. The change in lifestyle, increased participation of women in outdoor works, increased self-dependent individuals, increased workload and lack of time coupled with increased income, buying capacity, and status concerns has shifted the home cookery to institutional, restaurant, supermarket ready to eat products. Processing has also opened new avenues for value-added lost cost products development. In this regard utilization of edible by-products as, filler has a promising future. But for their adequate utilization the effect on textural and color characteristic is essential as texture and color are the two most important quality cues ascertaining consumer perception of meat and meat products [9].

The optimum utilization of edible byproducts has become essential for sustaining meat industry, reducing the environmental pollution and for alleviating nutritional scarcity. Comprehensive knowledge of these byproducts is required in respect of their functional properties for optimum utilization and maximizing returns to the meat industry sector. Scientific literature on the nutritional value of offals is relatively scarce and very few data are available on “variety meat and by-products” [10]. The various aspects of product development with edible byproducts or organ meat have been studied by some of the workers like [11-14]. But the textural, color and microbiological status and characteristics of the organs used has been unexplored.

Thus, the present study was designed with the objectives of determining the percentage yield of different edible by-products in goats; evaluating the microbiological quality of edible byproducts and for analyzing the textural and color characteristics of these by-products.

## Materials and Methods

### Ethical approval

The study was conducted after the approval of Institutional Animal Ethics Committee.

The work was conducted in Department of Livestock Products Technology, College of Veterinary Sciences and Animal Husbandry, DUVASU, Mathura, Uttar Pradesh, India and at Goat Products Technology (GPT) Laboratory, Central Institute for Research on Goats (CIRG), Makhdoom, Mathura, Uttar Pradesh, India. Twenty Barbari kids were reared in Barbari experiment unit at CIRG after weaning under routine conditions like grazing plus concentrate supplementation. Regularly animal was taken out for 8 h browsing daily in the grazing land of CIRG and concentrate mixture containing crude protein 14%, total digestible nitrogen 60% was also provided at the rate of 2% of body weight along with free access to clean drinking water twice daily, in morning before taking the animals out for grazing/feed offer and in the afternoon, on their return from grazing area. Ten male weaner Barbari kids each of 5-7 months of age having similar body conditions were used for the study. The selected animals were slaughtered as per the standard procedure at the experimental slaughterhouse of GPT laboratory of CIRG, after 16-18 h fasting with *ad-libitum* supply of water. Dressed carcasses were weighed within 1 h (hot carcass weight) and slaughter by-products were hygienically collected and weighed within 30 min longissimus muscle from loin cut (high value) was also collected and stored for further study as a reference in experimental studies. All the collected samples were immediately shifted to low-density polyethylene film pouches of 250 gauge thickness of natural color and stored at −18°C for further study.

### Carcass characteristics

#### Percent live weight

The weight of the organs and reference muscle was recorded to find out their contribution to the carcass on live weight basis.


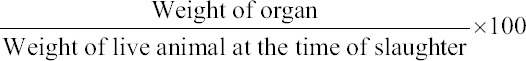


#### Percent carcass weight

The weight of the organs and reference muscle was recorded to find out their contribution to the carcass on carcass weight basis.


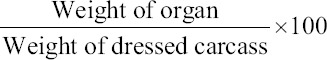


#### Texture profile analysis (TPA)

Textural properties of organ pieces were evaluated using the texturometer (stable micro system TA.XT-2i/25) at GPT Laboratory of Central Institute for Research on Goats (CIRG) Makhdoom, Mathura. TPA was carried out as per the method described by Bourne [15] to get the shear force and work of shear. Shear force (N/cm^2^) and work of shearing (Ns) of samples were estimated with Warner-Bratzler reversible blade attached to the same texture analyzer. Six 1 cm^3^ cylindrical bores were made at different positions on the organs, and these bores were used for analysis. The crosshead speed was 2 mm/s. Maximum force required to cut the sample (shear force) and the work needed to move the blade through the samples (work of shearing) were recorded.

#### Instrument color measurement

The color parameters of the edible by-products used in the study were monitored by Hunter ‘L*’, ‘a*’ and ‘b*’ values using color tech PCM+ (Color Tec Associates Inc. Clinton NJ, USA) and Chroma and hue values were derived from them.

#### Assay for microbiological quality

Total viable count (TVC), total coliform counts (CC), *Staphylococcus* spp. counts (SCC) in the samples were enumerated following the methods as described by American Public Health Association [16].

### Statistical analysis

The data obtained in the study on various parameters were statistically analyzed on “SPSS-19.0” Software Package as per standard methods [17]. For each parameter duplicate samples were drawn and were analyzed thrice (n=6). Data obtained were subjected to one-way analysis of variance, the homogeneity test and Duncan’s Multiple Range Test for comparing the means to find the effects between samples. The statistical significance was expressed at (p<0.05).

## Results and Discussion

The average age of animals studied was 188 days. While the average live weight of animals recorded during the research period was 17.5±0.44 kg and average carcass weight was 8.12±0.39 kg which was lower than the values reported by [18]. The average dressing percentage recorded was 46.38%. This result is in agreement with the findings of Das and Rajkumar [19] who reported a dressing percent of 45.11 in Barbari goats. But a higher dressing percentage was reported by Prpic *et al*. [20] which might be attributed to the fact that kidneys, kidney and pelvic fat were retained in carcass and was calculated together for the dressing percentage calculations. In a study Rajkumar *et al*. [21] have reported that for higher yield of variety meats younger animals under intensive system of management are better.

The percent live weight and carcass weight of different edible by-products have been tabulated in Table-1. The mean percent live weight as well as percent carcass weight was found to be highest in liver among all edible by-products. Similar findings were reported by Ockerman and Basu [22] who found the highest percent live weight for liver among all other edible by-products. The percent live weight of liver differed significantly (p<0.05) from those of spleen and brain while it evinced a non-significant (p>0.05) difference from that of kidney, heart, and testicles. The percent live weight of liver obtained during study in consort with the studies of Sen *et al*. [23] who reported the liver percent pre-slaughter weight as 1.32%, and Owen and Norman [24] observed average liver weight of 1.6% on live weight basis in Botswana kids. Prpic *et al*. [20] reported a little higher (1.86%) value for liver in the Croatian multicolored goat.

**Table-1:** Least square means (±SE) of percent live weight and percent carcass weight of different edible by-products of Barbari kids

Edible by-products	Liver	Kidney	Heart	Spleen	Brain	Testicles
Percent live weight (%)	1.47^a^±0.07	0.26^ab^±0.01	0.41^ab^±0.03	0.18^b^±0.01	0.16^b^±0.02	0.69^ab^±0.04
Percent carcass weight (%)	3.57^a^±0.19	0.62^cd^±0.03	0.99^bcd^±0.07	0.45^d^±0.03	0.40^d^±0.04	1.70^b^±0.12

Values within rows with different superscripts are significantly different (p<0.05), SE=Standard error

The percent live weight and the percent carcass weight of kidney and heart were in agreement with [25,26]. Percent live weight of spleen was 0.18% while percent carcass weight was 0.45. It was in agreement to Prpic *et al*. [20], Babiker *et al*. [27] who reported that percent spleen weight in goats of Sudan desert was 0.2-0.3%. Percent live weight of brain was found to be 0.164% in present study which was slightly lower than the values reported by Okerman and Basu [22] which could be attributed to the fact that the animals used in the study were of growing stage. The respective mean percent live weight and carcass weight of testicles was 0.69% and 1.70%. The percent live weight of testicles was higher than that of reported by Prpic *et al*. [20] and Mushi *et al*. [28] which may be because of breed or age of animals used for the study.

### Textural characteristics

The results of texture analysis have been presented in Table-2. The maximum (121.48±0.43) shear force and work of shear was evinced by liver while the minimum (3.48±0.11) values for the same was observed in brain. The textural properties of fresh meat are evaluated by number of muscle fibers and thickness of connective tissue septa which is measured by texture profile analysis. The Shear force value of muscle was within the range reported by Arguello *et al*. [29] i.e., 32.6-56.34 N but was lower than the values reported by [30]. The mean shear force and work of shear area values of liver and spleen were significantly higher (p<0.05) than that of muscle this could be attributed to their structural conformation. Liver is an organ composed mainly of parenchymatous tissue arranged into laminae associated with dense vasculature along with the connective tissue capsule that further divides the organ into lobes and lobules. Similarly, the spleen is covered with a capsule of dense irregular connective tissue oriented circumferentially. Various collagenous cords or trabeculae extend radially from the capsule into the body of spleen. These could be a possible reason for the higher shear force and work of share area values in liver and spleen. The Shear force value of kidney was non-significant (p>0.05) from that of muscle which could be due to its muscular structure quite similar to that of muscle. The Shear force value of brain was significantly (p<0.05) lower than other organs meat due to the soft structure and higher fat content.

**Table-2:** Textural characteristics of edible by-products and longissimus dorsi muscle of Barbari kids (means±SE)

Edible by-products	Muscle	Liver	Kidney	Heart	Spleen	Brain	Testicles
Shear force (N)	45.51^d^±0.62	121.48^a^±0.43	45.63^d^±0.59	57.81^c^±1.33	108.85^b^±0.99	3.49^f^±0.11	14.59^e^±0.13
Work of shear area (kg-sec)	19.90^d^±0.19	32.20^a^±0.19	17.34^e^±0.28	22.09^c^±0.34	26.95^b^±0.21	0.56^g^±0.02	6.61^f^±0.07

Values within rows with different superscripts are significantly different (p<0.05), SE=Standard error

The textural studies revealed that organ meats had varied textural characteristics. Spleen and liver showed higher shear force and work of shear than muscles which might be attributed to their higher connective tissue contents. Kidney and heart exhibited similar textural characteristics to muscles. Thus, these can be efficiently utilized in comminuted meat products. Brain evinced very low shear force and work of shear values. Thus, percent incorporation of these organs in various meat products needs to be further studied for their optimum level of incorporation without affecting the textural properties of the product.

### Color characteristics

**Figure-1:**
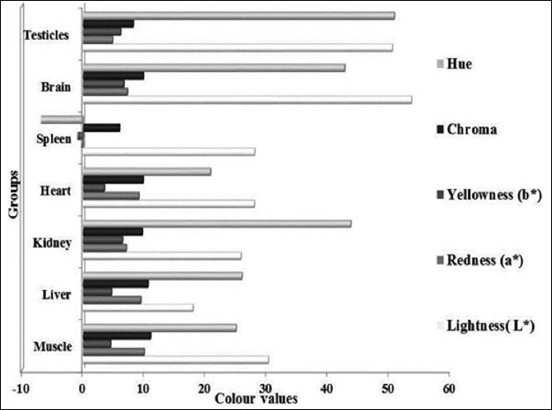
Instrumental color characteristics of edible by-products and longissimus dorsi muscle of Barbari kids (means ± standard error)

Color is one of the most important cue influencing the purchasing behavior of consumers [31]. The Hunter color values of *longissimus* muscle and edible by-products viz. liver, kidney, heart, spleen, brain and testicles were evaluated in terms of lightness (L*), redness (a*), yellowness (b*), chroma, and hue. The statistical values thus obtained have been presented in the Figure-1. The L*, a* and b* values of *longissimus* muscle in the present study were somewhat lower than the values reported by Pena *et al*. [32]; Kadim *et al*. [33]; Hussain *et al*. [34] which could be due the difference in the average age of animals studied in various experiments. The muscle color becomes darker with the progression of maturity in goats [32].

Brain and testicles showed lightness value in a higher range than that of muscle, while values of the rest of the organs lied in a lower range. Except for heart and spleen, all organs differed significantly (p<0.05) in their mean lightness values. The mean lightness and yellowness values were found to be highest in brain tissue among all organs meat which might be due to higher fat content resulting into higher reflectance. Kidney, heart, and spleen had closer mean lightness value to the muscle while brain and testicles evinced higher L* value this could be attributed to their higher moisture and fat content which cause higher reflectance.

The yellowness value of kidney, brain, and testicles was significantly (p<0.05) higher than that of muscles which could be attributed to their higher moisture contents. The redness value of liver differed non-significantly (p>0.05) from muscle while rest all organs showed significant (p<0.05) differences from it. The heart evinced a higher mean redness value than other organs meat due to its higher myoglobin and oxidative enzymes contents and even to the extensive blood supply whereas the redness value of spleen lied in the negative axis that indicated that the reflectance from the surface was the green axis instead of the red coordinate of chromaticity.

Chroma of all organs except liver differed significantly (p<0.05) from muscle, while hue of muscle, liver and heart differed non-significantly. The chroma and hue of longissimus dorsi muscle were in consort with the findings of [35].

The color attributes varied significantly among organs. Liver, kidney, and heart exhibited values close to that of muscles which imply that their incorporation in products would not much alter the color values of meat products. Spleen can be utilized to provide dark reddish color to the products as it evinced lower lightness value and inclination toward blue and green values.

### Microbiological assay

The microbiological status of freshly collected edible by-products was analyzed by estimation of total plate count (TPC), CC, SCC and the Salmonella count (log_10_ CFU/g). The mean and standard error values thus obtained are presented in the Table-3. The mean TPC were found to be significantly higher (p<0.05) in the liver as compared to other edible by-products. The possible reason behind this might be due to higher glycogen content which could be a good energy source for the growth of microorganisms. The initial TPC of edible offals was in order of 10^4^ log CFU/g. Patterson and Gibbs [36] also reported an initial APC of 10^4^-10^5^ CFU cm^−2^ for liver, kidney, heart, tongue, and diaphragm. The count in the present study were within the safe range as prescribed by CFR is 10^6^ CFU/g [37]. Abdullah [38] reported similar findings for the TVC count as observed in the present study.

**Table-3:** Microbial counts (log10 CFU/g) in different edible by-products and longissimus dorsi muscle of Barbari kids

Parameters/sample	Muscle	Liver	Kidney	Heart	Spleen	Brain	Testicles
TPC (Log CFU/g)	4.44^b^±0.03	4.85^a^±0.01	4.36^bc^±0.03	4.33^bc^±0.03	4.79^a^±0.01	3.90^d^±0.17	4.15^c^±0.13
Coliforms (Log CFU/g)	2.22^a^±0.12	2.32^a^±0.05	1.99^ab^±0.12	2.09^ab^±0.12	2.16^ab^±0.13	1.85^b^±0.12	2.14^ab^±0.09
*Staphylococcus* (Log CFU/g)	1.86^ab^±0.14	2.21^a^±0.13	1.98^ab^±0.11	2.06^ab^±0.12	2.11^a^±0.12	1.69^b^±0.13	1.85^ab^±0.14
Salmonella (Log CFU/g)	ND	ND	ND	ND	ND	ND	ND

Values within rows with different superscripts are significantly different (p<0.05), ND=Not detected, TPC=total plate count

The mean Staphylococcal count was found within the range of 1.97-2.24 which is acceptable raw organ meats [38]. The mean SCC of liver and spleen differed significantly (p<0.05) from that of the brain. The CC were in log 2 range which was also reported by [39]. The mean CC (log_10_ CFU/g) of liver and muscle differed significantly (p<0.05) from that of the brain. Rest of the organs differed non-significantly. Salmonella count was not at all detected in the present study, which possible reason behind this might be the good manage mental practices, hygienic handling, and processing of the organs during analysis.

## Conclusion

Edible by-products have a significant contribution to carcass weight which could increase total edible components of the carcass. If properly utilized, these can be a good source of revenue to the producers. Textural and color characteristics studied can serve as a guide for their incorporation in comminuted meat products. Further studies regarding cholesterol level are required before use of these edible byproducts in product formulations especially for byproducts like brain. Percent incorporation also requires detailed studies as to find out the adequate replacement percent of each byproduct which would not have a negative effect on physicochemical, nutritive, textural, and color characteristics of the end product. Studies regarding the residual drugs or chemicals should also be evaluated before commercializing these organs. The greatest hurdle in the efficient utilization of these byproducts is lack of infrastructure and modernized abattoirs designed with facilities for collection and storage of byproducts. Use of effective packaging for long and better-keeping quality of edible organs needs attention. The study was a preliminary and basic step forward toward better utilization of 3% of live animal that is yet not utilized to its fullest potential causing loss of 6.94% revenue (according to local market price of offals in Mathura).

## Authors’ Contributions

VP, VR, AKV designed the experiment. PU carried out the research work and prepared the manuscript with support of VPS. Manuscript was drafted and edited by ArKV. The final manuscript was read and approved by all the authors.
